# Supplementary material for: TET enzymes regulate skeletal development through increasing chromatin accessibility of RUNX2 target genes
Source: Nat Commun. 2022 Aug 11;13:4709. doi: 10.1038/s41467-022-32138-x (PMC9372040; doi:10.1038/s41467-022-32138-x)
Supplement: Supplementary file 1 — Supplementary Information [file 41467_2022_32138_MOESM1_ESM.pdf]

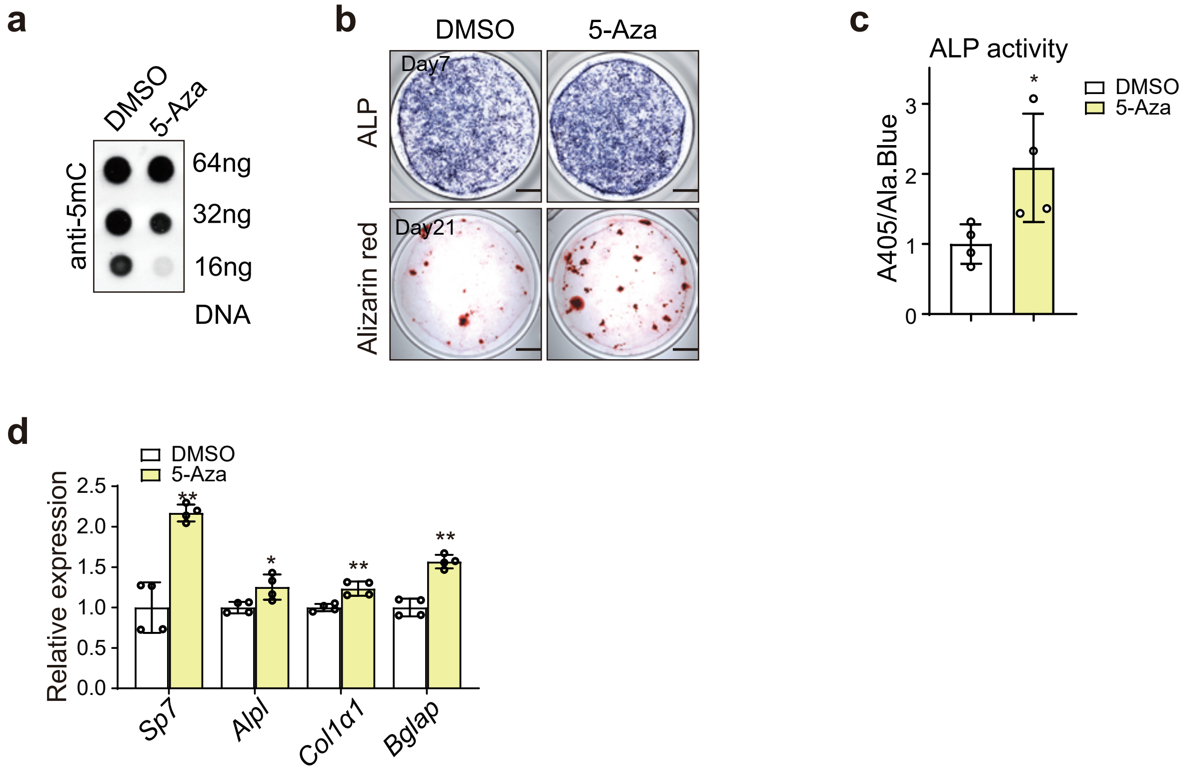

Supplementary Fig. 1. DNA hypomethylaton promotes osteoblast differentiation.
(**a**) Dot blot of 5m C in 1μM 5-Aza or control solvent -treated osteoblasts. The loading quantity of DNA was shown on the right as 16ng, 32ng and 64ng. (**b**) ALP staining and Alizarin red S staining after osteoblast differentiation for 7 days (top) and 21 days (bottom), respectively. Cells were treated with 1μM 5-Aza or control solvent. Scale bar = 1mm. (**c**) ALP activity quantification was measured by phosphatase substrate assay as A405/Ala.Blue. *P < 0.05 . Two-tailed Student's t-test. Data are presented as mean ± s.d., n = 4 independent cell supernatants. (**d**) RT-qPCR analysis of *Sp7*, *Alpl*, *Col1α1* and *Bglap* expression after osteoblast differentiation for 7 days. *P < 0.05, **P < 0.01. Two-tailed Student's t-test. Data are presented as mean ± s.d., n = 4 independent samples.

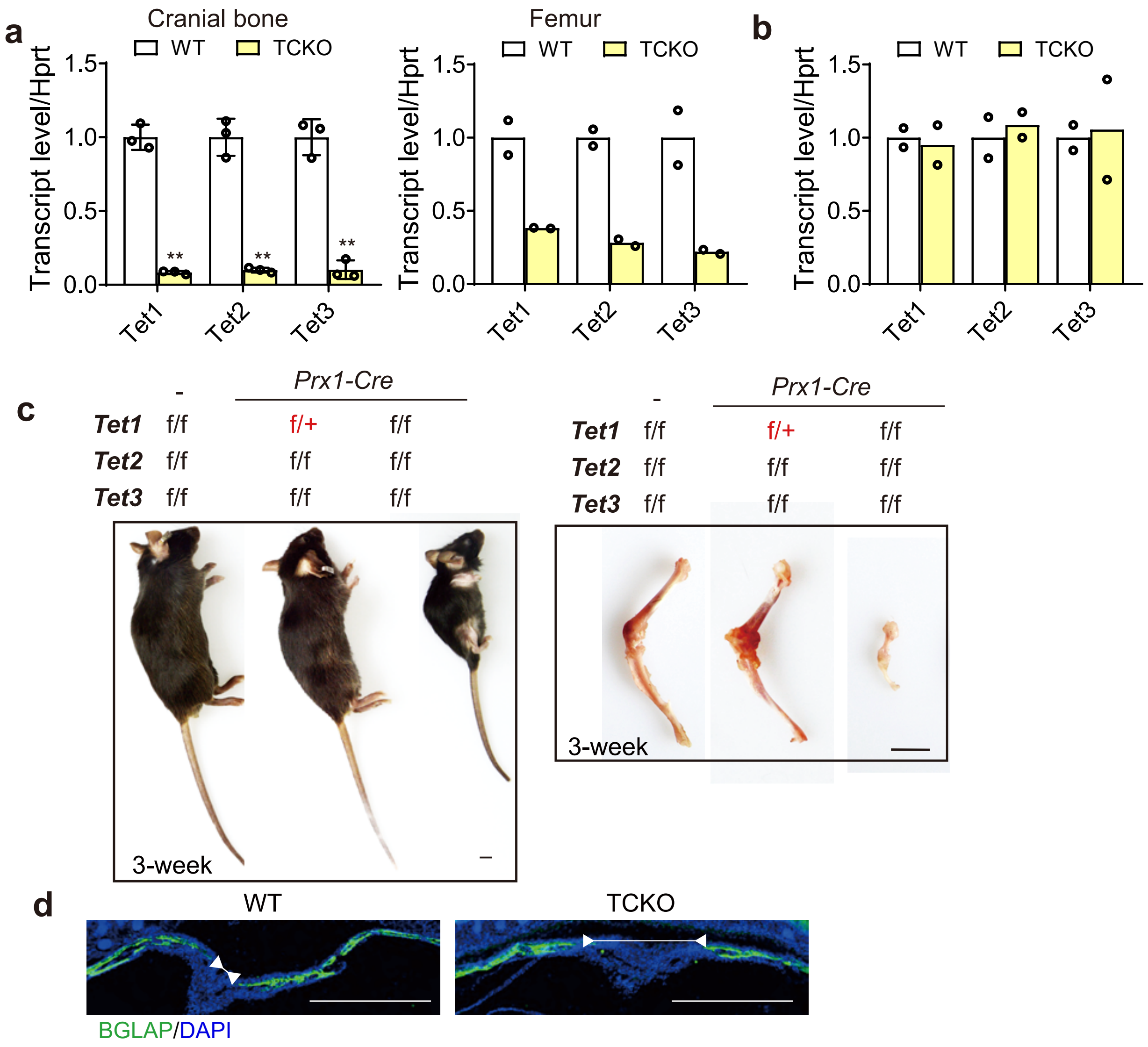

Supplementary Fig. 2. Loss of TET enzymes cause dwarfism in mice.

(**a-b**) RT-qPCR analysis of *Tet1*, *Tet2* and *Tet3* expression in cranial bones (**a, left**) and femurs (**a, right**), and liver (**b**) of WT and TCKO mice. **P < 0.01. Two-tailed Student's t-test. Data are presented as mean ± s.d., cranial bone: n = 3 biologically independent animals; femur: n = 2 biologically independent animals; liver: n = 2 biologically independent animals. (**c**) Gross images of indicated genotype mice for whole mice or limbs at 3 weeks. Scale bar = 5 mm. (**d**) Immunofluorescence of bone formation marker Osteocalcin (BGLAP) in the cranial bones from WT and TCKO mice at P0. Scale bar = 500μm. The lines with endpoint indicated the unmineralized region in the calvarial bone. Representative images for 2 independent samples.

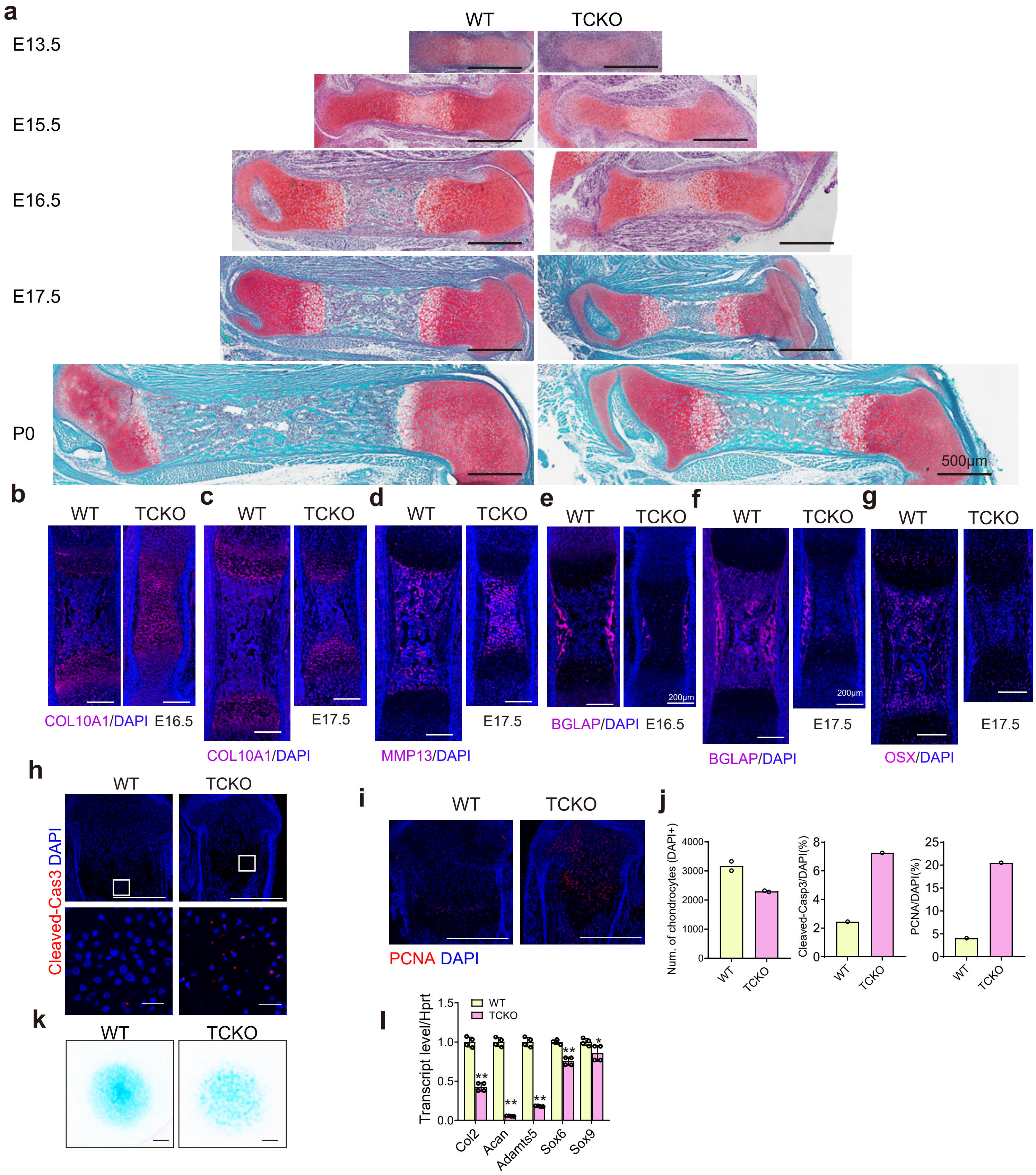

Supplementary Fig. 3. Loss of TET enzymes disrupt both the intramembranous and endochondral bone formation.

(**a**) Safranin O staining of limbs isolated from WT and TCKO mice at different developmental stages. Scale bar = 500μm. Representative images for 2 independent samples. (**b-g**) Immunofluorescence of COL10A1 at E16.5 (**b**) and E17.5 (**c**), MMP13 at E17.5 (**d**), BGLAP at E16.5 (**e**) and E17.5 (**f**), and OSX (**g**) in the femurs of WT and TCKO mice . Scale bar = 200μm. Representative images for 2 independent samples. (**h**) Immunofluorescence of cleaved-caspase 3 in the femurs of WT and TCKO mice at P0. Scale bar = 500μm for the top images and 40μm for the bottom images. (**i**) Immunofluorescence of proliferation marker PCNA ir the femurs of WT and TCKO mice at P0. Scale bar =500μm. (**j**) Quantification of PCNA-positive cells (n=1), cleaved Cas3 -positive cells (n=1), and the number of total chondrocytes (n=2 biologically independent animals) in (**h-i**). (**k**) Alcian blue staining of chondrocyte differentiation at day 7Scale bar = 1mm (**l**) RT-qPCR analysis of indicated genes expression after chondrocyte differentiation at day 7.*P < 0.05, **P < 0.01. Two-tailed Student's t-test. Data are presented as mean ± s.d., n = 4 independent samples.

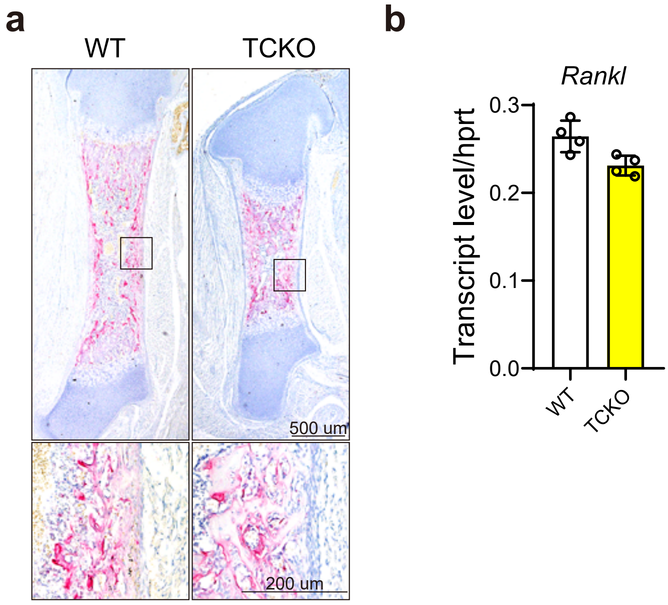

Supplementary Fig. 4. Loss of TET enzymes have no significant effects on bone remodeling after birth.

(**a**) TRAP staining in the femurs of WT and TCKO mice at P0. Scale bar= 500μm for the top images and 200μm for the bottom images. (**b**) RT-qPCR analysis of *Rankl* expression in the long bones of WT and TCKO mice.

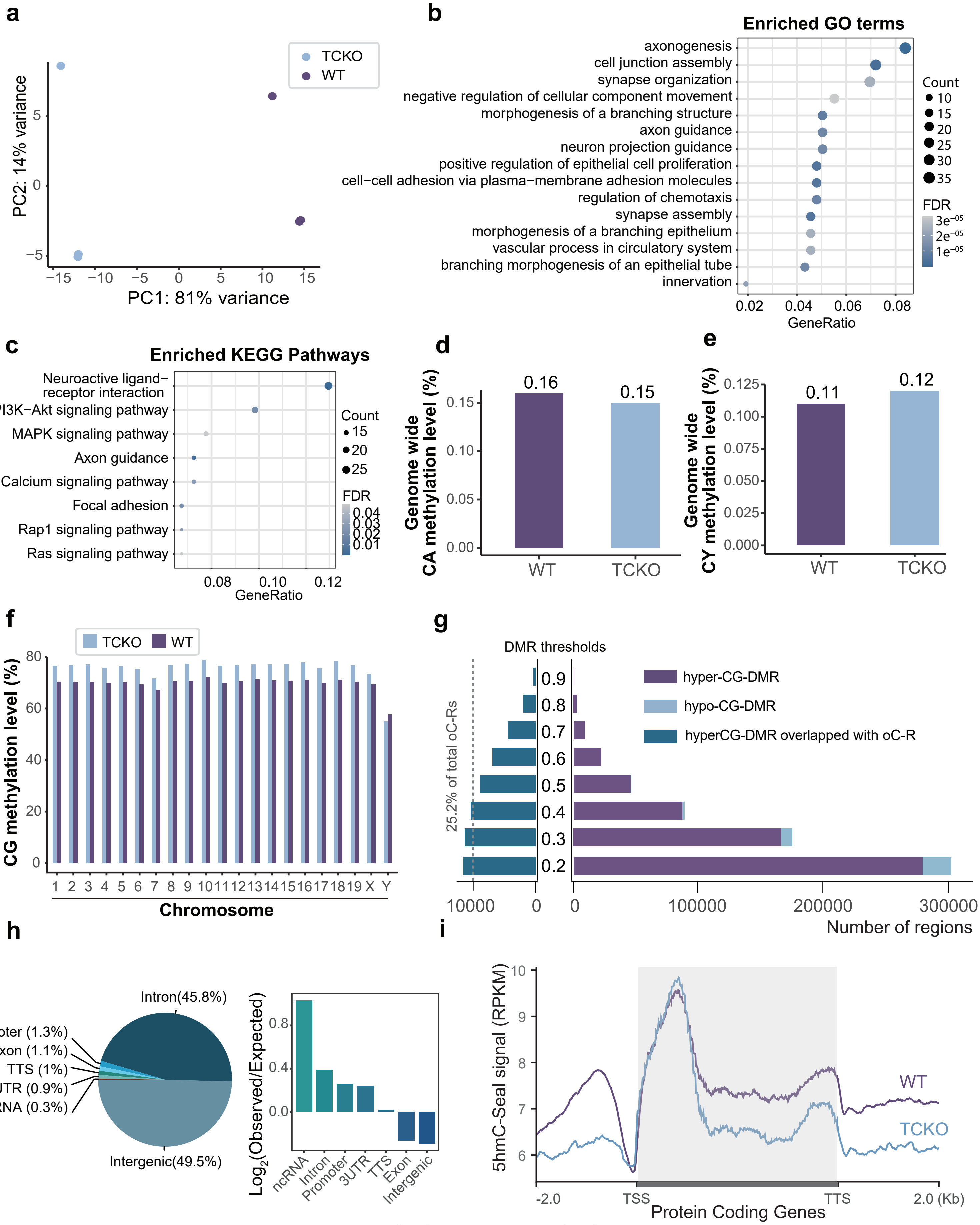

Supplementary Fig. 5. RNA-seq data and WGBS analysis in TCKO and WT cells, related to Figure 4. (**a**) PCA analysis of RNA-seq data of TCKO and WT cells over top 500 variable genes. (**b-c**) Enriched GO terms (**b**) and KEGG pathways (**c**) of up-regulated genes in TCKO cells. (**d-e**) Genome-wide CA and CY methylation level of TCKO and WT cells. (**f**) CG methylation level of TCKO and WT cells over each chromosome. (**g**) Number of differential methylated region (DMR) and overlapped regions of hyper-CG-DMR and Open-to-Close-Region (oC-R) in TCKO cells with different DMR thresholds. (**h**) Genomic distribution of hyper-CG-DMR in TCKO cells with pie and bar chart. (**i**) Metaplot of 5hmC signal over protein coding genes in TCKO and WT cells. ncRNA: no coding RNA, UTR: untranslated regions, TTS: transcription termination site, TSS: transcription start site.

a b

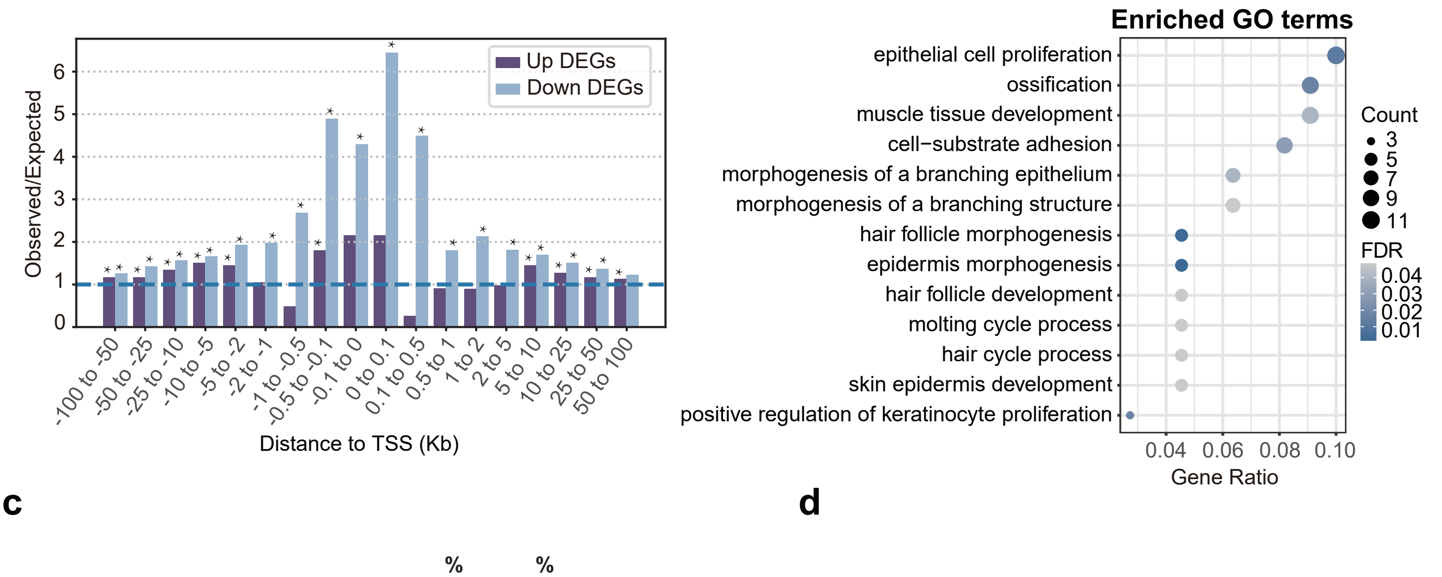

c

Motifs	P-value	% Target	% Background	Potential TFs
bZIP	1e-1072	11.16%	4.35%	Fra1/Fra2/Atf3/BATF/JunB/Fosl2/Ap-1/Jun-AP1/Bach2
Runt	1e-831	15.97%	8.87%	Runx1/Runx/Runx2/Runx-AML
CTF	1e-658	21.15%	13.73%	NF1-halfsite/NF1
TEA/TEAD	1e-519	16.59%	10.66%	TEAD3/TEAD1/TEAD/TEAD4
Homeobox,bHLH	1e-408	4.35%	1.84%	Pitx1:Ebox

d

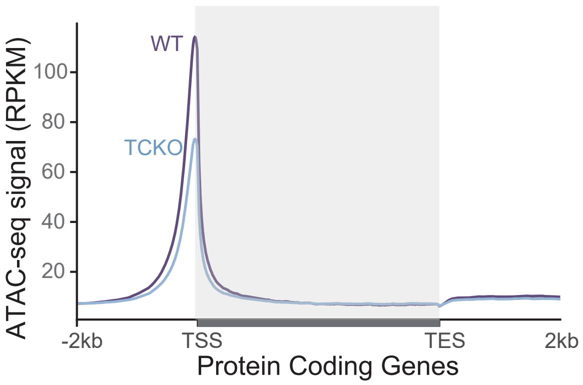

e f

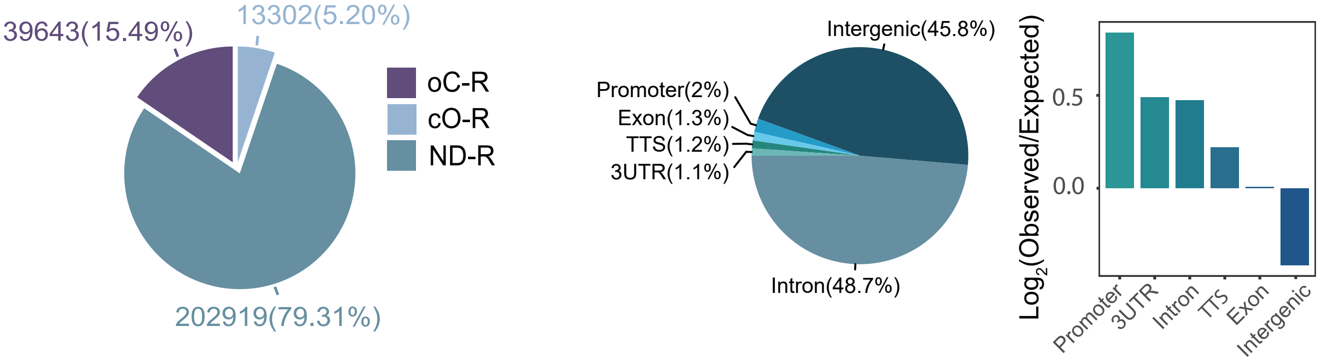

g

Motifs	P-value	%Target	%Background	Potential TFs
CTF	1e-560	30.65%	19.08%	NF1-halfsite/NF1
Runt	1e-545	18.55%	9.61%	RUNX1/RUNX2/RUNX-AML
Homeobox	1e-315	14.40%	8.17%	Six2
bZIP	1e-304	9.93%	4.92%	Atf3/BATF/Fra1/Fos/JunB/Fra2
TEA/TEAD	1e-296	16.69%	10.13%	TEAD3/TEAD1/

Supplementary Fig. 6. Associated analysis of RNA-seq, WGBS, and ATAC-seq data, related to Figure 5.

(**a**) Region associated DEG (RAD) analysis for regions shared by hyper-CG-DMR and DEGs in TCKO cells compared with WT.* P < 0.01; hypergeometric test. (**b**) Enriched GO term of down regulated genes inTCKO cells, which had a distance less than 2kb from hyper-CG-DMR. (**c**) Top five enriched known motifs and their potential transcription factors in hyper -CG-DMR in TCKO cells; hypergeometric test. (**d**) Metaplot of ATAC peaks signal over protein coding genes i n TCKO and WT cells. (**e**) Pie chart of chromatin Open -to-Close-Region (oC -R), Close -to-Open-Region (cO -R) and Non -Difference-Region (ND -R) in TCKO cells. (**f**) Genomic distribution of TCKO oC -R with pie and bar chart. (**g**) Top five enriched known motifs and th eir potential transcription factors in TCKO oC -R; hypergeometric test . UTR: untranslated regions, TTS: transcription termination site.

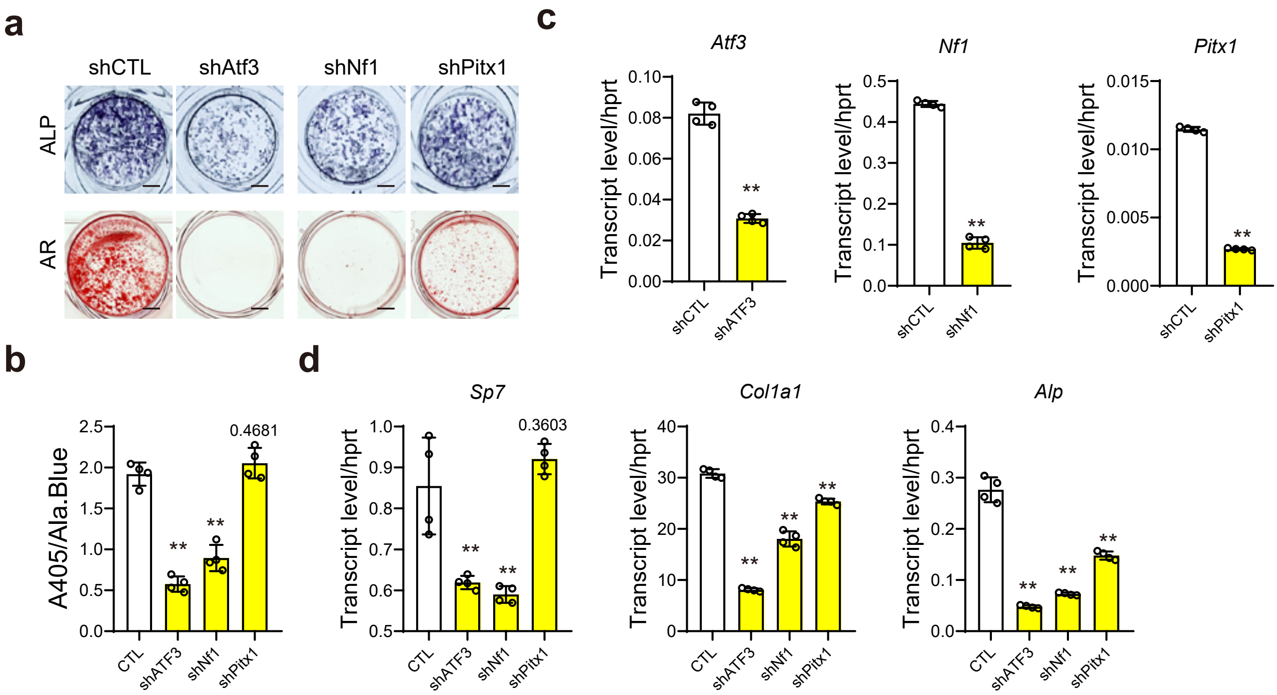

Supplementary Fig. 7. The function of the predicated transcription factors on osteoblast differentiation.

(**a**) ALP staining and Alizarin red S staining after osteoblast differentiation for 7 days (top) and 21 days (bottom), respectively. Scale bar = 1mm. (**b**) ALP activity quantification was measured by phosphatase substrate assay as A405/Ala.Blue. **P < 0.01. Two-tailed Student's t-test. Data are presented as mean ± s.d., n=4 independent cell supernatants. (**c-d**) RT-qPCR analysis of indicated gene expression after osteoblast differentiation for 7 days.**P < 0.01. Two-tailed Student's t-test. Data are presented as mean ± s.d., n = 4 independent samples.

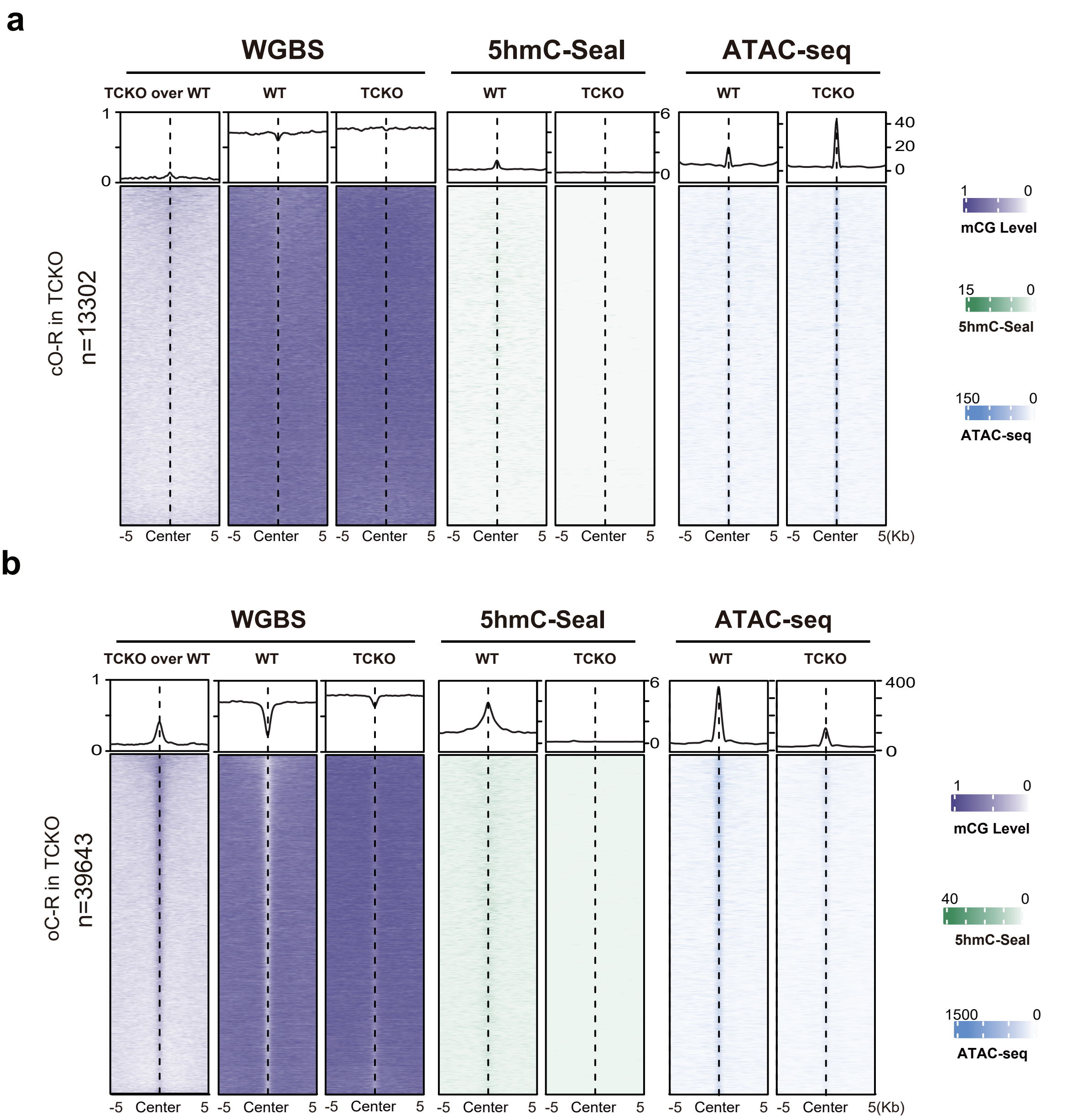

Supplementary Fig. 8. Associated analysis of RNA -seq, WGBS, 5hmC -Seal and ATAC -seq data, related to Figure 5.

(**a-b**) Heatmap and metaplot of CG methylation level, 5hmC-Seal and ATAC-seq signal over chromatin Close-to-Open-Region (cO-R) (**a**) and Open-to-Close-Region (oC-R) (**b**) in TCKO cells.

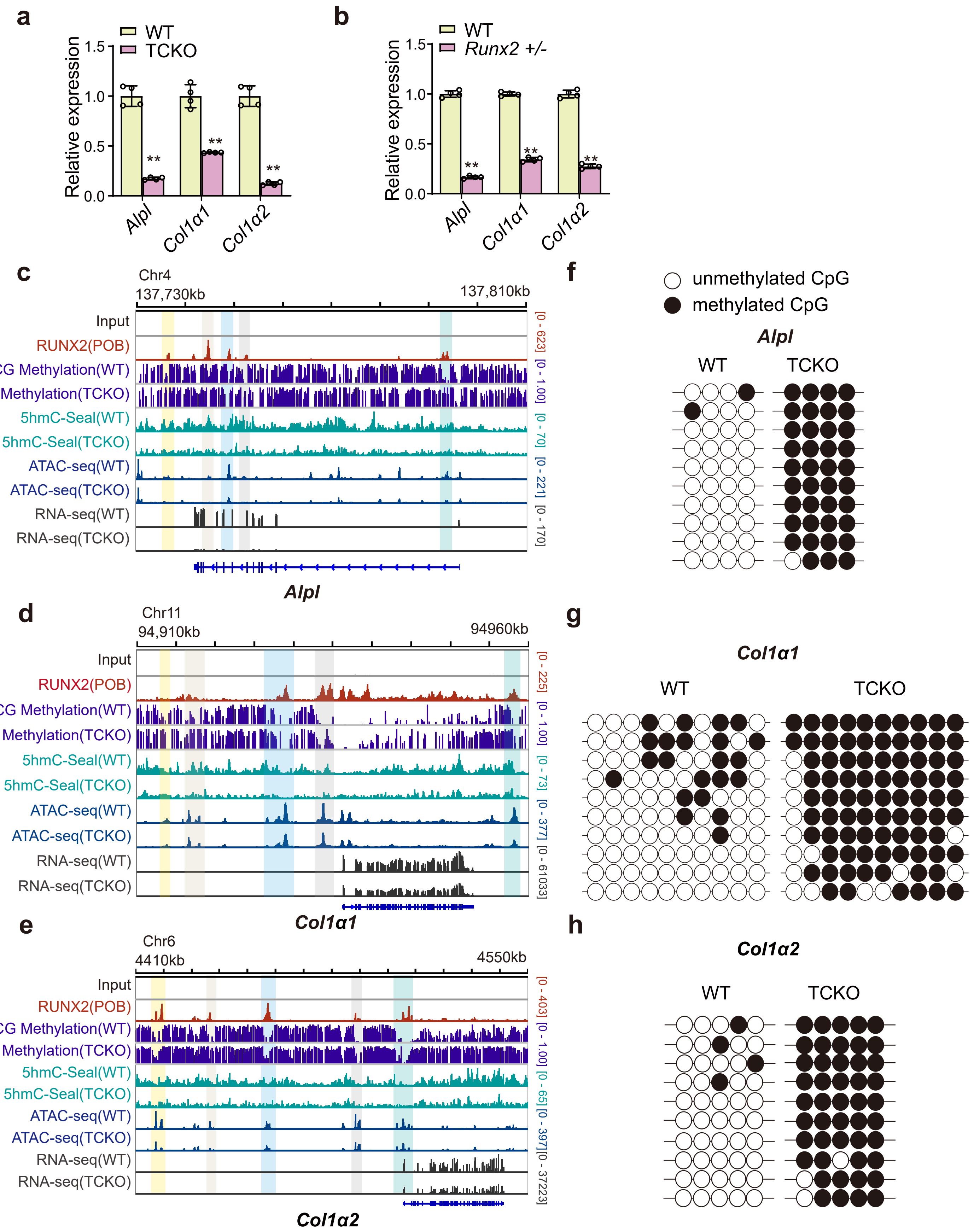

Supplementary Fig. 9. RUNX2 binding sites co-localize with TET-dependent hyper-CG-DMR.
(**a-b**) The expression pattern of *Alpl*, *Col1α1*, *Col1α2* in the primary osteoblasts isolated from WT and TCKO mice (**a**) and from WT and *Runx2+/-* mice (**b**). *P < 0.05, **P < 0.01. Two-tailed Student's t-test. Data are presented as mean ± s.d., n = 4 independent samples. (**c-e**) Screenshot of indicated genes from RUNX2 ChIP-seq and input in pre-osteoblastic MC3T3-E1 cells (pre-osteoblast, POB) (GSM1027478), CG methylation level from WGBS, 5hmC-Seal, ATAC-seq, and RNA-seq in WT and TCKO primary osteoblasts over osteogenic marker genes including *Alpl* (**c**), *Col1α1* (**d**), and *Col1α2* (**e**) genes. Number with the brackets indicate the relative levels for ChIP-seq, WGBS, 5hmC-Seal, ATAC-seq and RNA-seq data. (**f-h**) Methylation analysis of *Alpl* (**f**), *Col1α1* (**g**), *Col1α2* (**h**) in the primary osteoblasts isolated from WT and TCKO mice by bisulfite Sanger sequencing. The open and black circles represent the unmethylated and methylated CpG sites, respectively.

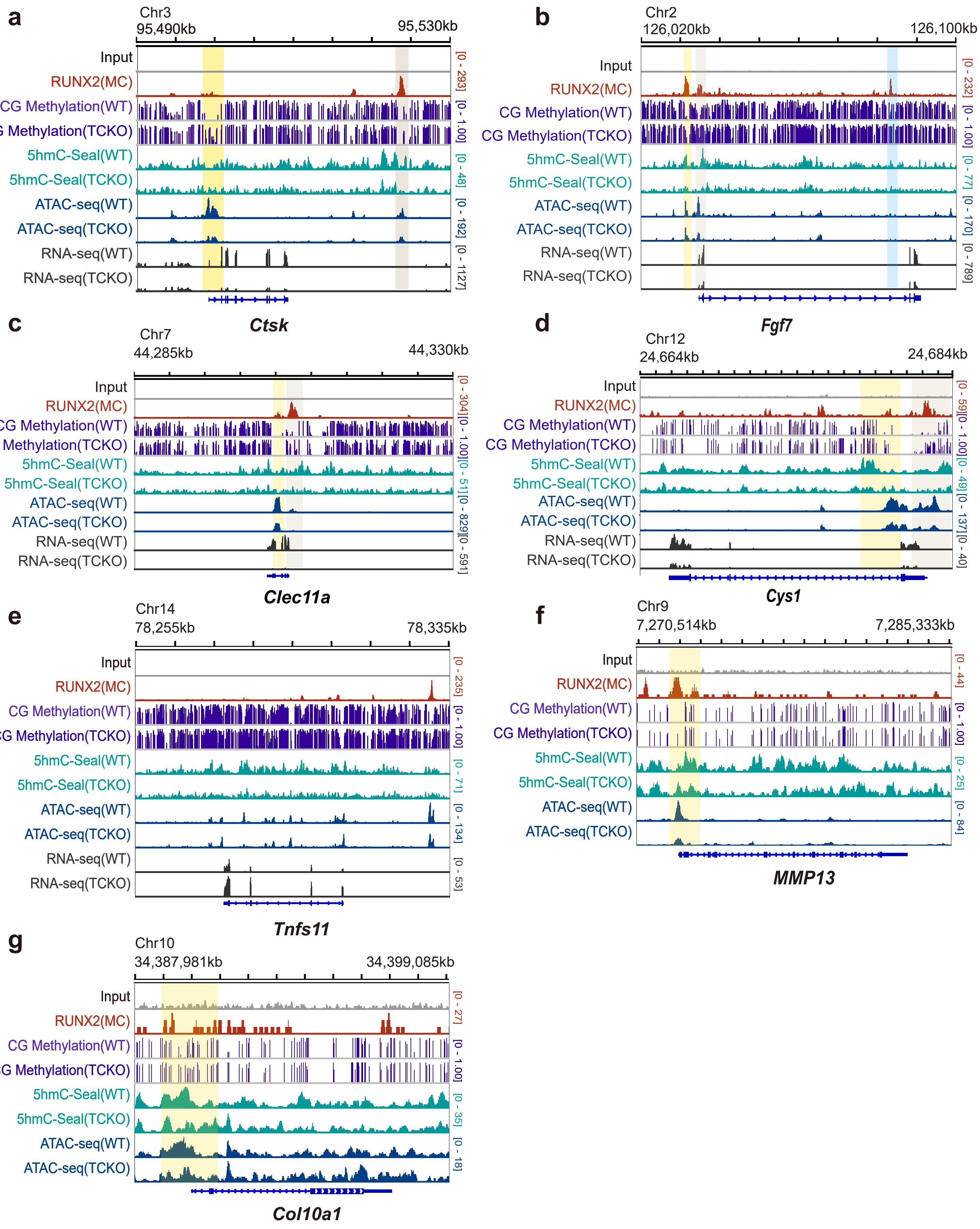

Supplementary Fig. 10. RUNX2 binding sites co-localize with TET-dependent hyper-CG-DMRs. (**a-g**) Screenshot of RUNX2 ChIP-seq and input in pre-osteoblastic MC3T3-E1 cells (MC) (GSM1027478), CG methylation level from WGBS, 5hmC-Seal, ATAC-seq, and RNA-seq in WT and TCKO primary osteoblasts over genes including *Ctsk* (**a**), *Fgf7* (**b**), *Clec11a* (**c**), *Cys1* (**d**), *Tnfs11* (**e**), *Mmp13* (**f**) and *Col10a1* (**g**) genes. Number with the brackets indicate the relative levels for ChIP-seq, WGBS, 5hmC-Seal, ATAC-seq and RNA-seq data.

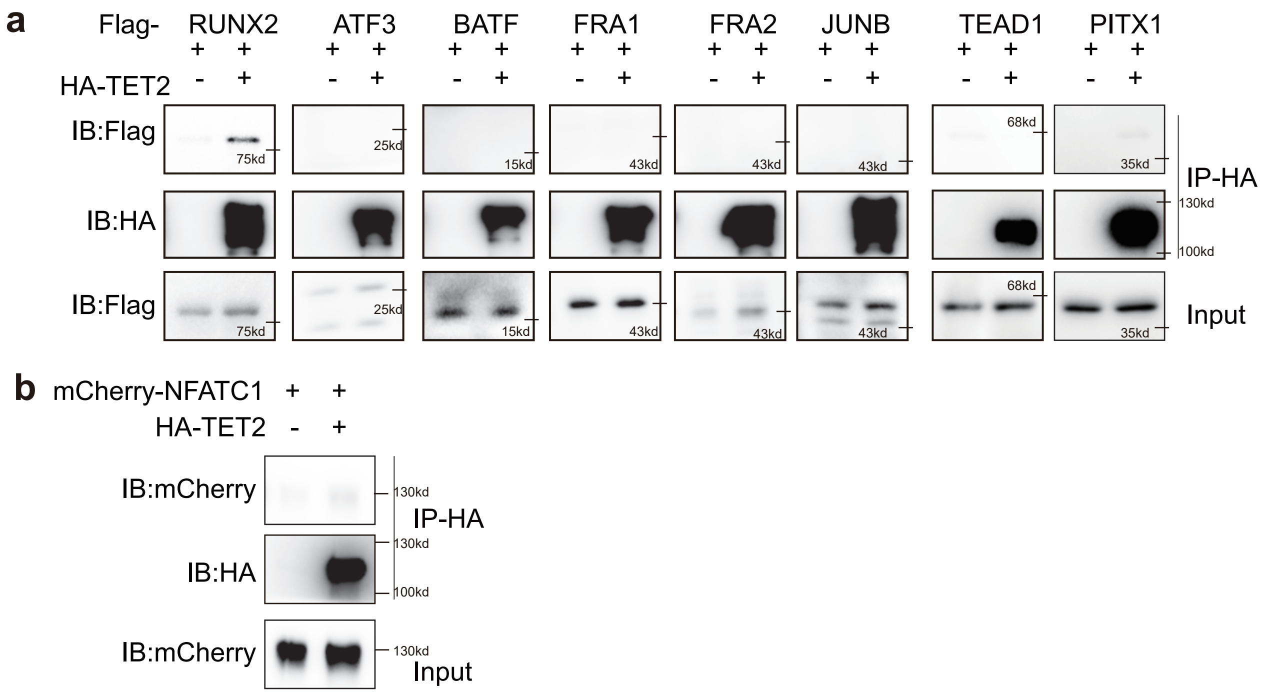

Supplementary Fig. 11. Screening of the potential transcriptional factors interacted with TET proteins. (**a-b**) Co-immunoprecipitation (Co -IP) of HA-TET2 with indicated transcriptional factors in 293T cells. Representative images for 2 independent samples.

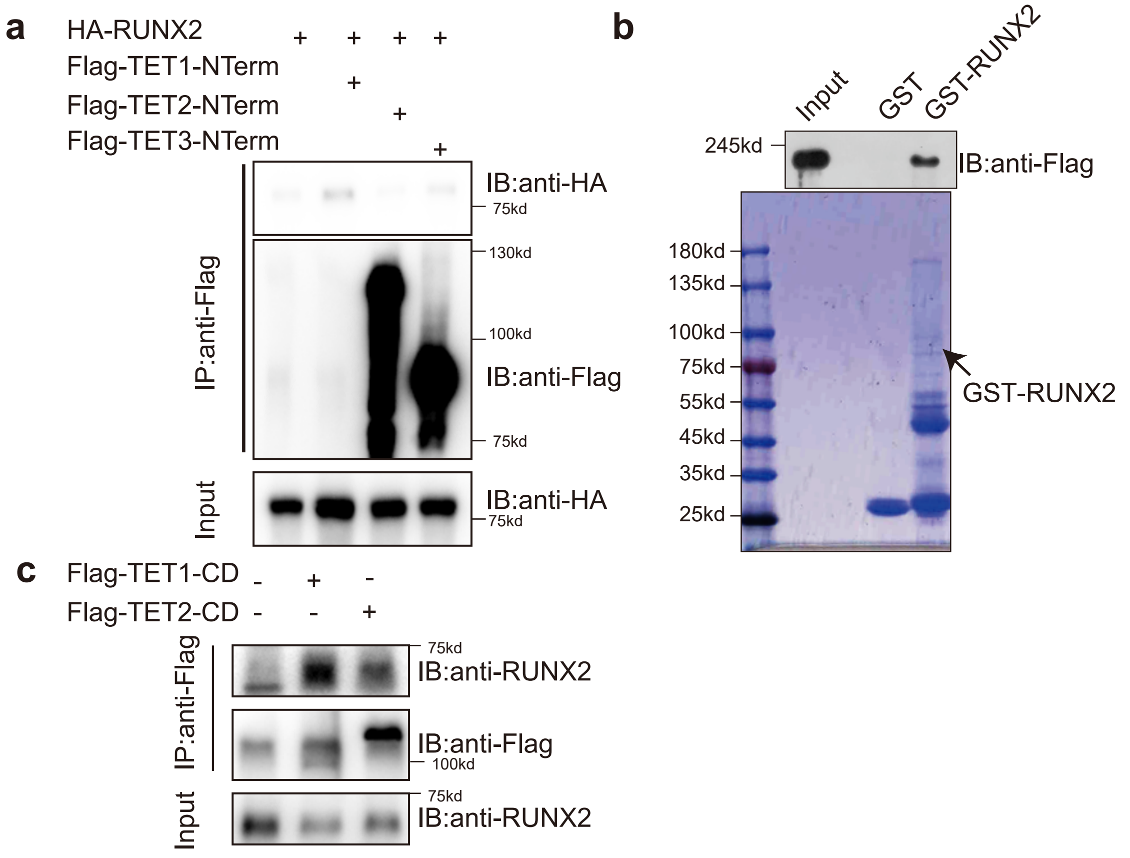

Supplementary Fig. 12. Interaction between TET proteins and RUNX2.

(**a**) Co-immunoprecipitation (Co-IP) of Flag-TET1-Nterm or Flag-TET2-Nterm or Flag-TET3-Nterm with HA-RUNX2. HA-RUNX2 expressing plasmid was co-transfected with Flag-TET1-Nterm or Flag-TET2-Nterm or Flag-TET3-Nterm in 293T cells. Whole cell lysate was used for immunoprecipitation and then blotted with indicated antibodies. (**b**) Flag-TET2-CD expressed in 293T waspurified by Flag beads, followed by incubatio glutathione S-transferase (GST) or GST -RUNX2. The bound proteins were precipitated with glutathione agarose, followed by western blotting with indicated antibod y. (**c**) Co-immunoprecipitation (Co-IP) of Flag-TET1-CD or Flag-TET2-CD with endogenous RUNX2 in C3H10T1/2 cells. Flag-TET1-CD or Flag-TET2-CD lentivirus was infected the C3H10T1/2 cells. Whole cell lysate was used for immunopr ecipitation with Flag-Beads and then blotted with indicated antibodies. Representative images for 2 independent samples for **a**-**c**.

Supplementary Table 1. hyper CG methylation with DEGs in TCKO cells					
SYMBOL	type_of_TCKO_DEG	Distance_To_Nearest_Sites	SYMBOL	type_of_TCKO_DEG	Distance_To_Nearest_Sites
1500015O10Rik	dw	-1kb	Asb2	dw	-1kb
Cavin2	dw	-1kb	Card6	dw	-1kb
Fmod	dw	-1kb	Pik3ap1	dw	-1kb
Dhrs9	dw	-1kb	Kcne4	dw	0kb
Fgf7	dw	-1kb	Fmod	dw	0kb
Tnmd	dw	-1kb	Sec16b	dw	0kb
Ctsk	dw	-1kb	Fgf7	dw	0kb
Adh7	dw	-1kb	Cass4	dw	0kb
Col10a1	dw	-1kb	Tnmd	dw	0kb
Slc7a2	dw	-1kb	S100a4	dw	0kb
Sorbs2	dw	-1kb	Ctsk	dw	0kb
Sh2d4a	dw	-1kb	Adh7	dw	0kb
Dok2	dw	-1kb	Tlr4	dw	0kb
Plscr2	dw	-1kb	Runx3	dw	0kb
Gngt2	dw	-1kb	Slc22a18	dw	0kb
Agtr1a	dw	-1kb	Avil	dw	0kb
Ecm2	dw	-1kb	Anxa8	dw	0kb
Dglucy	dw	-1kb	Prss35	dw	0kb
Ifi27	dw	-1kb	Plscr2	dw	0kb
Rpl39l	dw	-1kb	Ogn	dw	0kb
Mylk	dw	-1kb	Sostdc1	dw	0kb
Plekhh2	dw	-1kb	Tnn	dw	0kb
Dock8	dw	-1kb	Fap	dw	0kb
Mr1	dw	-1kb	Gpr155	dw	0kb
Tnn	dw	-1kb	Rtl3	dw	0kb
Fap	dw	-1kb	Veph1	dw	0kb
Rtl3	dw	-1kb	Gimap6	dw	0kb
Slc26a7	dw	-1kb	Clec11a	dw	0kb
Per3	dw	-1kb	Fcgrt	dw	0kb
Eln	dw	-1kb	Emp3	dw	0kb
Fam180a	dw	-1kb	Phactr2	dw	0kb
Gimap6	dw	-1kb	Calhm5	dw	0kb
Zim1	dw	-1kb	Glipr1	dw	0kb
Fcgrt	dw	-1kb	Gpr183	dw	0kb
Gpr183	dw	-1kb	Cys1	dw	0kb
Nnmt	dw	-1kb	Rspo2	dw	0kb
Evi2a	dw	-1kb	Krt80	dw	0kb
Itga1	dw	-1kb	Col8a1	dw	0kb
Lrrn3	dw	-1kb			
