# Supplementary material for: TET enzymes regulate skeletal development through increasing chromatin accessibility of RUNX2 target genes
Source: Nat Commun. 2022 Aug 11;13:4709. doi: 10.1038/s41467-022-32138-x (PMC9372040; doi:10.1038/s41467-022-32138-x)
Supplement: Supplementary file 3 — Reporting Summary [file 41467_2022_32138_MOESM3_ESM.pdf]

nature portfolio

Corresponding author(s): Weiguo Zou

Last updated by author(s): Jul 8, 2022

Reporting Summary

Nature Portfolio wishes to improve the reproducibility of the work that we publish. This form provides structure for consistency and transparency in reporting. For further information on Nature Portfolio policies, see our Editorial Policies and the Editorial Policy Checklist.

Statistics

For all statistical analyses, confirm that the following items are present in the figure legend, table legend, main text, or Methods section.

n/a	Confirmed	
☐	☒	The exact sample size (*n*) for each experimental group/condition, given as a discrete number and unit of measurement
☐	☒	A statement on whether measurements were taken from distinct samples or whether the same sample was measured repeatedly
☐	☒	The statistical test(s) used AND whether they are one- or two-sided<br>*Only common tests should be described solely by name; describe more complex techniques in the Methods section.*
☒	☐	A description of all covariates tested
☒	☐	A description of any assumptions or corrections, such as tests of normality and adjustment for multiple comparisons
☐	☒	A full description of the statistical parameters including central tendency (e.g. means) or other basic estimates (e.g. regression coefficient) AND variation (e.g. standard deviation) or associated estimates of uncertainty (e.g. confidence intervals)
☐	☒	For null hypothesis testing, the test statistic (e.g. *F*, *t*, *r*) with confidence intervals, effect sizes, degrees of freedom and *P* value noted<br>*Give P values as exact values whenever suitable.*
☒	☐	For Bayesian analysis, information on the choice of priors and Markov chain Monte Carlo settings
☒	☐	For hierarchical and complex designs, identification of the appropriate level for tests and full reporting of outcomes
☒	☐	Estimates of effect sizes (e.g. Cohen's *d*, Pearson's *r*), indicating how they were calculated

Our web collection on statistics for biologists contains articles on many of the points above.

Software and code

Policy information about availability of computer code

Data collection	GraphPad Prism (8.0.1), image J (1.53k), BSMAP (v 2.74), STAR(v2.7.0e), Fastqc(v0.11.8), Cutadapt(v2.3), Samtools(v1.2), bamCoverage (v3.3.1), featureCounts(v2.0.0), macs2(2.1.6), Homer(V4.10.4), bedtools(v2.29.0), Deptools(V3.3.1), R(4.0.6)
Data analysis	1.1 RNA-seq analysis<br>The RNA sequencing reads were aligned to GRCm38.99 Mus musculus reference genome with STAR(v2.7.0e) using a supplied set of known transcripts in GTF format (RefSeq GRCm38.99; Mus musculus, Ensembl). Differentially expressed genes were calculated using DESeq2 with cutoff (Fold change greater than 2, FDR less than 0.05, and RPKM greater than 0.24 - the first quartile), and RPKM values were calculated with a custom R script. Once differential expressed genes were determined, R package clusterProfiler was used for further analysis of GO terms and KEGG pathway.<br>1.2 WGBS analysis<br>For WGBS data analysis, raw reads were aligned to the reference GRCm38.99 Mus musculus genome using BSMAP (v 2.74) by allowing up to 2 mismatches (-v 2), 1 best hit (-w 1) and aligning to both strands(-n 1). Methylation levels at each cytosine were then extracted with BSMAP (methratio_32unit_alt.py) scripts by allowing only unique mapped reads (-u). Methylation levels at each cytosine were calculated as #C/(#C +#T). DMRs were then defined with R package DMRcaller over the whole genome at least 4 CG sites in 200 bins and minimal CG methylation level difference greater than 40% and P-value less than 0.05 and min Gap as 100bp. In order to identify the genomic distribution of hyper-CG-DMR and predominant motifs in hyper-CG-DMR, HOMER(v4.10.4) was applied to 200 bp around the middle points of hyper-CG-DMRs. The hyper-CG-DMRs associated with DEGs analysis (RAD) was was performed with the RAD web application via https://labw.org/rad to calculate the distance between hyper -CG-DMR and DEGs.<br>1.3 ATAC-seq analysis<br>ATAC-seq data were aligned to the reference GRCm38.99 Mus musculus genome using STAR(v2.7.0e) by using EndToEnd alignEndsType and allowing only uniquely mapping reads with fewer than three mismatches and only 1 for maximum intron length. Peaks were called using MACS2 with call-summits method and other default parameters (v 2.1.6). To find peaks specific to one condition (e.g. TCKO), specific peaks to each state were defined with a two-fold relative enrichment cutoff after merging the replicates. Genomic distribution and motif analysis of

specific peaks in TCKO was calculated using HOMER (v4.10.4) with 200 bp around the middle points of peaks. To determine the overlapped regions of oC-R with hyper-CG-DMRs, we used the bedtools(v2.29.0) intersect tool.
1.4 ChIP-seq and 5hmC-Seal analysis
For ChIP-seq and 5hmC-Seal data analysis, raw reads were aligned to the reference GRCm38.99 Mus musculus genome using STAR(v2.7.0e) with the same parameters of ATAC-seq analysis. Peaks were called using MACS2 with call-summits method and other default parameters (v 2.1.6). ChIP-seq and 5hmC-Seal metaplots and heatmaps were generated using R package Enriched Heatmap.
2. IMAGING ANALYSIS for quantification of immunofluorescence positive cells: image J (1.53k)
3. Statistical analysis was performed using GraphPad Prism 8 software (8.0.1)

For manuscripts utilizing custom algorithms or software that are central to the research but not yet described in published literature, software must be made available to editors and reviewers. We strongly encourage code deposition in a community repository (e.g. GitHub). See the Nature Portfolio guidelines for submitting code & software for further information.

Data

Policy information about availability of data

All manuscripts must include a data availability statement. This statement should provide the following information, where applicable:

- Accession codes, unique identifiers, or web links for publicly available datasets
- A description of any restrictions on data availability
- For clinical datasets or third party data, please ensure that the statement adheres to our policy

All data and genetic material used for this paper are available from the authors on request. RNA-seq, WGBS, 5hmC-Seal, and ATAC sequencing data generated in this study have been deposited at NCBI GEO under accession number GSE174048 and is publicly available. Runx2 ChIP-seq data used in Figure 5.d-e and Supplementary Figure 9 and 10 is from a published dataset in GEO under accession GSM1027478 (https://www.ncbi.nlm.nih.gov/geo/query/acc.cgi?acc=GSM1027478) (Meyer, M.B., Benkusky, N.A. & Pike, J.W. The RUNX2 cistrome in osteoblasts: characterization, down-regulation following differentiation, and relationship to gene expression. J Biol Chem 289, 16016-31 (2014). Source data for Figures and Supplementary Figures have been provided in the Source Data File in this paper.

Field-specific reporting

Please select the one below that is the best fit for your research. If you are not sure, read the appropriate sections before making your selection.

☒ Life sciences ☐ Behavioural & social sciences ☐ Ecological, evolutionary & environmental sciences

For a reference copy of the document with all sections, see nature.com/documents/nr-reporting-summary-flat.pdf

Life sciences study design

All studies must disclose on these points even when the disclosure is negative.

Sample size	No statistical methods were used to predetermine sample size. Sample sizes were decided based on the literature and prior experience in our laboratory (doi: 10.1172/JCI132518; doi: 10.1126/sciadv.aba4147.).
Data exclusions	No data were excluded in this study.
Replication	For animal-based experiment, we will use at least 3 animals in one group. For cell based experiment, it will be replicated for at least twice. All attempts at replication were successful.
Randomization	Samples and mice were randomly allocated to different groups. Mice used in this study were age- and sex-matched in the same experiment.
Blinding	For animal-based experiment, mice were blinded to group allocation. For cell based experiment, investigators were not blind to the experiments as investigators were not able to influence the outcome of the experiment. The same protocols and criteria were used to determine the difference between control and experimental group in independent experiments and the results can be well replicated.

Reporting for specific materials, systems and methods

We require information from authors about some types of materials, experimental systems and methods used in many studies. Here, indicate whether each material, system or method listed is relevant to your study. If you are not sure if a list item applies to your research, read the appropriate section before selecting a response.

Materials & experimental systems

n/a	Involved in the study
	☒ Antibodies
	☒ Eukaryotic cell lines
☒	Palaeontology and archaeology
	☒ Animals and other organisms
☒	Human research participants
☒	Clinical data
☒	Dual use research of concern

Methods

n/a	Involved in the study
☒	ChIP-seq
☒	Flow cytometry
☒	MRI-based neuroimaging

Antibodies

Antibodies used

goat-anti-OPN (R&D, AF808, BDO0720111, 1:1000;), rabbit-anti-BGLAP (ABCAM, ab93876, GR3357172-2,1:200), rabbit-anti-RUNX2 (ABCAM, ab236639, GR3388032-6,1:200), mouse-anti-5mC (ABCAM, ab10805, GR284756-4, 1:1000), rabbit-anti-5hmC (active motif, 39792,1:1000), rabbit-anti-Col10a1 (Abclonal, a6889, 3561636001, 1:200), rabbit-anti-Mmp13 (Proteintech, 18165-1-AP, 021, 1:200), rabbit-anti-Osx (ABCAM, ab22552, GR3357012-2, 1:200), biotin-anti-PCNA (Biolegend, 307904, B213618, 1:200), rabbit-anti-Cleaved-Caspase3 (CST, 9664T, 21, 1:200), Donkey-anti-mouse 488(Molecular Probes, A21202, 1:1000), Donkey-anti-goat 488 (Molecular Probes, A11055, 1:1000), donkey-anti-rabbit cy3 (Jackson ImmunoResearch, 711-165-152, 1:1000), APC-Streptavidin (BioLegend, 405207, B266711, 1:500), HA antibody (Santa Cruz Biotechnology, sc-7392, 1:2000),Flag antibody (Sigma, F3165-5MG, 1:5000), Anti-mouse-immunoglobulins/HRP (Abbkine, A2502, 1:2000),Pol II antibody (Millipore, 05-623, 3ug/1×10^6 cells),Normal IgG (Millipore, 12-371, 3ug/1×10^6 cells).

Validation

All antibodies used in this study were verified by the vendor.
goat-anti-OPN: https://www.rndsystems.com/cn/products/mouse-osteopontin-opn-antibody_af808
rabbit-anti-BGLAP: https://www.abcam.com/osteocalcin-antibody-ab93876.html
rabbit-anti-RUNX2: https://www.abcam.com/runx2-antibody-epr22858-106-chip-grade-ab236639.html
mouse-anti-5mC: https://www.abcam.com/5-methylcytosine-5-mc-antibody-33d3-ab10805.html
rabbit-anti-5hmC: https://www.activemotif.com/catalog/details/39791/5-hydroxymethylcytidine-antibody-pab
rabbit-anti-Col10a1: https://abclonal.com.cn/catalog/A6889
rabbit-anti-Mmp13: https://www.ptglab.com/products/MMP13-Antibody-18165-1-AP.htm
biotin-anti-PCNA: https://www.biolegend.com/en-us/products/biotin-anti-human-mouse-rat-pcna-antibody-813?GroupID=GROUP28
rabbit-anti-Osx: https://www.abcam.com/sp7--osterix-antibody-ab22552.html
rabbit-anti-Cleaved-Caspase3: https://www.cellsignal.com/products/primary-antibodies/cleaved-caspase-3-asp175-5a1e-rabbit-mab/9664
Donkey-anti-mouse 488: https://www.thermofisher.cn/cn/zh/antibody/product/Donkey-anti-Mouse-IgG-H-L-Highly-Cross-Adsorbed-Secondary-Antibody-Polyclonal/A-21202?adobe_mc=MCMID%7C36049315079583334622260415142316926429%7CMCAID%3D3060DD60EDF24520-40000807E808D2E4%7CMCORGID%3D5B135A0C5370E6B40A490D44%40AdobeOrg%7CTS=1614293705
donkey-anti-rabbit cy3: https://www.jacksonimmuno.com/catalog/products/711-165-152
Donkey-anti-goat 488: https://www.thermofisher.cn/cn/zh/antibody/product/Donkey-anti-Goat-IgG-H-L-Cross-Adsorbed-Secondary-Antibody-Polyclonal/A-11055?adobe_mc=MCMID%7C36049315079583334622260415142316926429%7CMCAID%3D3060DD60EDF24520-40000807E808D2E4%7CMCORGID%3D5B135A0C5370E6B40A490D44%40AdobeOrg%7CTS=1614293705
APC-Streptavidin: https://www.biolegend.com/en-us/products/apc-streptavidin-1470?GroupID=GROUP23
HA antibody: https://www.scbt.com/p/ha-probe-antibody-f-7
Flag antibody: https://www.sigmaaldrich.cn/CN/zh/product/sigma/f3165?gclid=Cj0KCQjwtvqVBhCVARIsAFUxcRuO0MSTP1uF_l_kDRtgrN2yD2s30axhKB9QnWQg0_MUsEr4Wu5yvxoaAkFfEALw_wcB
Anti-mouse-immunoglobulins/HRP: https://www.abbkine.com/product/hrp-goat-anti-mouse-igg-a21010/
Pol II antibody: https://www.sigmaaldrich.cn/CN/zh/product/mm/05623
Normal IgG: https://www.sigmaaldrich.cn/CN/zh/product/mm/12371

Eukaryotic cell lines

Policy information about cell lines

Cell line source(s)	293T cells(ATCC, CRL-3216), C3H10T1/2 cells(ATCC, CCL-226).
Authentication	This cell lines were used in our previous study and not authenticated in our lab.
Mycoplasma contamination	All cell lines tested negative for mycoplasma contamination.
Commonly misidentified lines (See ICLAC register)	No commonly misidentified cell lines were used in the study.

Animals and other organisms

Policy information about studies involving animals; ARRIVE guidelines recommended for reporting animal research

Laboratory animals

Tet1fl/fl Tet2fl/fl Tet3fl/fl mice and Tet1fl/HD Tet2fl/fl Tet3fl/fl mice (gifts from Guoliang Xu, Shanghai Institute of Biochemistry and

	Cell Biology) were crossed with the Prx1-Cre strain (a gift from Andrew McMahon, Harvard University) to generate Prx1-Cre, Tet1fl/fl Tet2fl/fl Tet3fl/fl mice, Prx1-Cre, Tet1fl/+ Tet2fl/fl Tet3fl/fl mice, Prx1-Cre, Tet1fl/fl Tet2fl/+Tet3fl/fl mice, Prx1-Cre, Tet1fl/fl Tet2fl/fl Tet3fl/+ mice, Prx1-Cre, Tet1fl/HD Tet2fl/fl Tet3fl/fl mice and Prx1-Cre, Tet1+/HD Tet2fl/fl Tet3fl/fl mice. All mice analyzed were maintained on the C57BL/6 background. Animals were euthanized by CO_2. The experiments were performed with new born mice or embryonic mice. The specific detailed age was noted in the figure legend. Animals were bred and maintained under specific pathogen free (SPF) conditions with the 12/12 hours light/ dark cycle, 25℃ and 45%-65% humidity in the institutional animal facility of the Shanghai Institute of Biochemistry and Cell Biology, Chinese Academy of Sciences.
Wild animals	The study did not involve samples collected from the field.
Field-collected samples	The study did not involve samples collected from the field.
Ethics oversight	All animal experiments were performed with protocol approved by the Animal Care and Use Committee of Shanghai Institute of Biochemistry and Cell Biology, Chinese Academy of Sciences.

Note that full information on the approval of the study protocol must also be provided in the manuscript.
